# Imaging Polarized Secretory Traffic at the Immune Synapse in Living T Lymphocytes

**DOI:** 10.3389/fimmu.2018.00684

**Published:** 2018-04-06

**Authors:** Víctor Calvo, Manuel Izquierdo

**Affiliations:** Departamento de Bioquímica, Instituto de Investigaciones Biomédicas Alberto Sols CSIC-UAM, Madrid, Spain

**Keywords:** T lymphocytes, immune synapse, secretory granules, multivesicular bodies, exosomes, cytotoxic activity, cell death

## Abstract

Immune synapse (IS) formation by T lymphocytes constitutes a crucial event involved in antigen-specific, cellular and humoral immune responses. After IS formation by T lymphocytes and antigen-presenting cells, the convergence of secretory vesicles toward the microtubule-organizing center (MTOC) and MTOC polarization to the IS are involved in polarized secretion at the synaptic cleft. This specialized mechanism appears to specifically provide the immune system with a fine strategy to increase the efficiency of crucial secretory effector functions of T lymphocytes, while minimizing non-specific, cytokine-mediated stimulation of bystander cells, target cell killing and activation-induced cell death. The molecular bases involved in the polarized secretory traffic toward the IS in T lymphocytes have been the focus of interest, thus different models and several imaging strategies have been developed to gain insights into the mechanisms governing directional secretory traffic. In this review, we deal with the most widely used, state-of-the-art approaches to address the molecular mechanisms underlying this crucial, immune secretory response.

## Introduction

### Immune Synapse and Polarized Secretory Traffic

**Figure 1:**
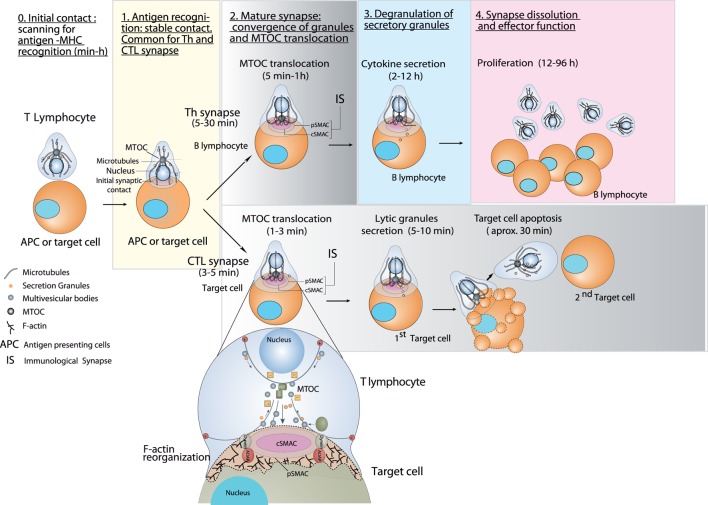
Stages of helper T (Th) and cytotoxic synapses and polarized secretion toward the IS. Stages 0 and 1 are common for both Th and cytotoxic T lymphocyte (CTL) synapses. After the initial scanning contact for specific antigen–major histocompatibility complex (MHC) complexes, Th effector T lymphocytes (upper chain of events) form mature synapses with antigen-presenting B lymphocytes within several minutes. This IS lasts many hours during which *de novo* cytokine (i.e., IL-2, IFN-γ) production (involving *de novo* gene transcription) and secretion occurs, that requires continuous T cell receptor (TCR) signaling. After IS formation, Th lymphocytes may also undergo non-polarized (multidirectional) secretory traffic of certain cytokines (TNF-α, IL-4) (13). This fact has not been depicted for clarity reasons. The cell conjugates split after several hours, and then the lymphocytes eventually proliferate. Primed effector CTLs (lower chain of events) establish more transient, mature synapses after scanning their target cells (i.e., a cell infected with a virus), and deliver their lethal hits within a few minutes. Secretory lysosomes (lytic granules) are very rapidly transported (within very few minutes) toward the microtubule-organizing center (MTOC) (in the minus “−” direction) and, almost simultaneously, the MTOC polarizes toward the central supramolecular activation cluster (cSMAC) of the IS, a filamentous actin (F-actin)-depleted area that constitutes a secretory domain (14). Non-polarized, multidirectional exocytosis of lytic granules from naïve CTLs has been shown also to be induced by resting human B cells (12). This fact has not been depicted for clarity reasons. MTOC translocation to the IS appears to be dependent of dynein motors anchored to adhesion and degranulation promoting adapter protein (ADAP) at the peripheral supramolecular activation cluster (pSMAC), that pull MTOC in the minus direction (15, 16). In both types of synapses (lower zoom panel), the initial F-actin reorganization in the cell–cell contact area, followed by a decrease in F-actin at cSMAC and an accumulation at the pSMAC appears to be involved in granule secretion (17, 18). Multivesicular bodies (MVBs) are also secretion granules involved in the polarized secretion of exosomes at the IS upon their degranulation (19, 20).

T cells can initially and transiently interact with antigen-presenting cell (APC) *via* accessory, adhesion molecules such as LFA-1 (1). This allows the lymphocyte to remain in close, but also labile, contact with APC and to scan the APC’s surface for specific antigen–major histocompatibility complex (MHC) complexes (2, 3). If the APC does not carry a specific antigen, then the T cell does not remain attached to the APC and can interact and examine other potential APCs for specific antigen (Figure 1) (3, 4). This trial-and-error characteristic is considered an important mechanism to assure the specific interaction of the T cell receptor (TCR) with specific antigen-bearing APCs (1, 3). When TCR encounters specific antigen on APC, a productive TCR stimulation by antigen presented on APC induces the formation of the immune synapse (IS) (5, 6). The formation of the IS constitutes an essential component of the immune system (6). IS comprises a highly ordered and plastic, signaling platform that integrates signals and coordinates molecular interactions leading to an appropriate and specific immune response (6). In T lymphocytes, once TCR encounters a specific antigen bound to MHC, one early consequence of IS formation constitutes the convergence of the secretory granules toward the microtubule-organizing center (MTOC) and, almost simultaneously, the polarization of the MTOC to the IS (7, 8) (Figure 1). Acting coordinately, these two trafficking events finely ensure the specificity of T cell effector responses, by enabling polarized secretory traffic toward the APC (7, 8), spatially and temporally focusing the secretion at the synaptic cleft (9). However, it should be pointed out that not always MTOC polarization is necessary or sufficient for lytic granule transport to the IS and cytotoxic hit delivery. In this context, it has been shown that an initial and rapid step of lytic granule secretion constitutively located nearby the IS precedes MTOC polarization at the cytotoxic T lymphocyte (CTL)/target cell synapse (10). In addition, it has been shown that PKCδ-deficient CTL efficiently reoriented MTOC in response to target cell recognition but were not able to polarize their lytic granules (11). These results broaden current views of CTL biology by revealing an extremely rapid step of lytic granule secretion and by showing that MTOC polarization is dispensable for efficient lethal hit delivery. Moreover, there is evidence that resting human B cells escape killing by CTLs by inducing non-polarized exocytosis of their lytic granules, although MTOC translocated normally toward the IS (12). Non-polarized degranulation was associated with an altered formation of the IS and may represent a mechanism that allows B cell malignancies to evade CTLs (12). These examples of segregation between MTOC movement and lytic granules traffic point out that the analyses of both MTOC repositioning and traffic of secretory vesicles should be considered to obtain the full picture of the secretion process.

### Two Kinds of Secretory Synapses

The outcome of the IS depends on the type of T lymphocyte and APC. The interaction of helper T (Th) cells (usually CD4+ cells) with the antigen-presenting, MHC-II-bearing APC results in the activation of the T cell (cytokine secretion, proliferation, etc.). In the case of CTLs (CD8+ cells) interacting with APC displaying antigen-associated MHC-I the response depends on the pre-stimulation of the CTL with the antigen. Thus, naïve CTLs recognizing antigen–MHC-I complexes on APC are “primed” to kill target cells and proliferate. Primed CTL also form IS with target cells (tumor cells or cells infected by viruses) resulting in specific killing (Figure 1). The functional changes produced by the establishment of a productive, mature IS include activation (for Th cells), activation (naïve CTL) or killing (primed CTL), apart from functional anergy or apoptosis (3). Thus, there are two major groups of secretory immune synapses made by T lymphocytes that lead to very different, but crucial immune effector functions (5, 6, 21). On one hand, IS made by primed CTL triggers the rapid polarization (from seconds to very few minutes) of secretory granules (called “lytic granules” or “secretory lysosomes”) toward the synapse (Figure 1). Degranulation of these lytic granules induces the secretion of perforin and granzymes to the synaptic cleft (22). In addition, relocalization of pro-apoptotic Fas ligand from the limiting membrane of the secretory lysosome to the cell surface occurs (23). Fas ligand on the CTL cell surface subsequently triggers apoptosis by binding to Fas-expressing target cells (24). Acting together, all these death-inducing factors trigger the death of the target cells (25). CTLs form somewhat transient synapses, lasting only few minutes, as the target cells are killed (8, 26). This is probably due to the fact that the optimal CTL function requires a rapid and transient contact to deliver as many lethal hits as possible to several target cells (8, 26) (Figure 1). On the other hand, Th lymphocytes make stable, lengthy synapses (>20–30 min up to several hours) that are necessary for both directional and continuous secretion of stimulatory cytokines (8, 26). The fact these Th synapses are more stable than the synapses formed by CTLs does not exclude the possibility that Th may establish sequential contacts with several APCs, as has been shown for B cells and dendritic cells (5, 27). These cytokines are contained in secretory granules, and some of them (IL-2, IFN-γ) also undergo polarized traffic to the IS (8). Accordingly, in long-lived synapses made by Th lymphocytes the MTOC takes from several minutes up to hours to move and dock to the IS, whereas in primed CTLs the directional movement of MTOC toward the synapse lasts very few minutes (6, 8, 26) (Figure 1). Considering these points, it becomes clear that a major difference between the IS established by primed CTL and that established by Th cells is the overall duration of the process and its immediate repeatability. To eliminate target cells rapidly, CTL contacts with target cells are quick, and CTLs can establish multiple IS with different target cells over short periods of time (8). Conversely, Th cells establish prolonged IS and do not rapidly form consecutive IS once activated properly (Figure 1) (4, 6). In any case, both the convergence of secretory granules toward the MTOC and MTOC polarization to the IS appear to be necessary for optimal polarized and focused secretion in many cell types of the immune system, including innate natural killer (NK) cells (28, 29), CTLs (17, 30), primary CD4+ T cells (31), and Jurkat cells (30), although some exceptions to this situation have been described in the first paragraph. This specialized mechanism appears to specifically provide the immune system with a finely tuned strategy to increase the efficiency of crucial secretory effector functions, while minimizing non-specific, cytokine-mediated stimulation of bystander cells target cell killing and activation-induced cell death (AICD).

### Polarized Secretion of Exosomes

Polarized secretion of exosomes at the IS constitutes an emerging and challenging field involved in relevant immune responses (32). In this context, it has been shown that late endosomes with multivesicular body (MVB) structure carrying intraluminal vesicles (ILVs) undergo polarized traffic toward the IS (Figure 1, lower panels) (19). The degranulation of MVBs at the IS induces the release of ILVs as exosomes to the synaptic cleft. This occurs in synapses formed by Jurkat cells, TCR-stimulated CD4+ lymphoblasts, and primed CTLs (19, 20, 33–37). While exosomes are constitutively secreted by various cell lineages and tumor cells, in T and B lymphocytes exosome secretion is triggered upon activation of cell surface receptors, which in turn regulates antigen-specific immune responses (25, 38). The exosomes participate in important processes related to TCR-triggered immune responses, including T lymphocyte-mediated cytotoxicity, AICD of CD4+ lymphocytes, antigen presentation, intercellular miRNA exchange (20, 35, 39–42), and thymus development (43). However, the mechanisms underlying MVB traffic and exosome secretion are poorly understood when compared with those corresponding to other secretory granules in T lymphocytes (21, 44, 45). Thus, the study of this partially unknown polarized secretion pathway using the proposed imaging techniques will provide information regarding an important, specialized function of the immune system.

## Methods for Immune Synapse Formation and Imaging

The formation of the IS consists of a highly rapid and plastic process that may conduct to several different polarized traffic outcomes, and some of these may differ in time. For instance, polarization of lytic granules of CTLs takes very few minutes, whereas certain cytokine-containing granules take from several minutes up to several hours to complete their polarization. These time differences need to be considered in advance to design the experiment and to choose the right experimental approach, since for some imaging strategies time is a limiting factor.

One important feature of the IS consists of the establishment of exploratory, labile contacts between the T cell and the APC, that may lead or not to a stronger contact and the formation of a mature synapse, providing that TCR recognizes the antigen–MHC complexes and the establishment of proper costimulatory interactions (3). Both the onset of the initial contacts and the establishment of a mature, fully productive IS, are intrinsically stochastic, rapid and asynchronous processes (3, 46). In addition, there is a low frequency in the formation of cell–cell conjugates (47). Another major challenge in studying polarization of secretory machinery in T lymphocytes, particularly in CTLs, is that the whole process is quite fast (a few minutes). For all these reasons, most early studies have used an end-point approach in which T lymphocytes and target cells are mixed together in suspension, concentrated by low speed centrifugation to favor conjugate formation, incubated for several minutes, fixed and scored for the repositioning of MTOC and/or secretory granules toward the IS (48). This approach has two important drawbacks: no dynamic trafficking information was obtained, and high levels of background MTOC/secretory granules polarization due to the stochastic nature of IS formation were observed (46). In addition, any correlation between TCR-derived early signaling events (i.e., rises in intracellular calcium, actin reorganization) and secretory granule polarization is difficult to analyze. Therefore, important pre-requisites for proper imaging of the IS in living cells include to increase the conjugate formation efficiency, to synchronize the formation of the IS and, if possible, to guarantee the formation of conjugates at certain microscope fields (*XY*) and focal plane (*Z*) positions. Several approaches have been designed to guarantee this synchronicity and to circumvent all these caveats. We will describe below some examples for these approaches, pointing out some of their advantages and weaknesses.

### Planar Lipid Bilayers and Coverslip/Beads-Coated With Surface Proteins or Agonistic Antibodies

This approach reduces a three-dimensional (3D) complex structure such an IS to two dimensions (*XY*) and enables high-resolution imaging (4). Using this strategy several relevant issues can be solved. On one hand, the synchronicity issue can be solved since cells will eventually sediment and will get into the microscope focus and stimulated at the coverslip interface with a similar time course. On the other hand, stimulation will occur at a homogenous, defined *Z* position; this will facilitate image capture. It is well known that conventional epifluorescence and confocal microscopes, but also super-resolution microscopes, have poor resolution in the *Z*-axis (49). Thus, by excluding *Z* dimension, specific high-resolution techniques such as total internal reflection microscopy (TIRFM) (see below) will benefit from this approach. Movement of secretory granules at the focus plane will consist of centripetal convergence toward the central supramolecular activation cluster (cSMAC) area (5, 50) and this can be recorded and analyzed (Figure 1). Unfortunately, this technique can be somewhat reductionist since it is not possible to guarantee that all the molecular interactions occurring in a cell–cell synapse will occur also upon interaction with the bilayer (5). In fact, supported lipid bilayers do not mimic the complex surface of an APC or target cell, giving rise to non-physiological interactions in the IS (51). In addition, capture in a defined *Z* in the very initial moments of the interaction with the coverslip will exclude intracellular structures (i.e., secretory vesicles) located beyond a certain *Z* distance from the coverslip (>200–300 nm). However, for some structures contained and reorganizing within the IS (i.e., actin reorganization), the features of some images obtained by using anti-TCR-coated coverslips and lipid bilayers and imaged by TIRF and TIRF–structured illumination microscopy (SIM) combination (see below) probably exhibit by far the highest quality obtained to date (50, 52). The possibility to define and change the composition of the lipid bilayer by loading antigens, accessory molecules, lipids, etc., allows for “reconstitution approaches” and offers flexibility to this approach (4).

However, the information obtained from *in vitro* bilayers systems must be considered with caution when extrapolated to *in vivo* T cell activation and synapse formation. In this context, the antigen or antigenic peptide dose affects the size of the cSMAC, and the levels of antigen–MHC on endogenous APCs may be lower than the levels used in the *in vitro* models (5). The rigid and flat nature of the glass-supported bilayer may also drive kinetic size segregation, and *in vitro* cell–cell conjugates tend to have multifocal and quite heterogeneous synapses (5). For instance, kinetic segregation of CD45 has been shown to be crucial for TCR triggering (53) and this may differently occur in T cells interacting with lipid bilayers with respect to cell–cell conjugates. Finally, indeed the APC develops mechanical and functional contributions to IS surfaces but also modulates synapse structure and function (5, 12). Thus, it is important to take into account that studies with supported planar bilayers are powerful in terms of resolution and sensitivity, but it is always important to test the predictions of these model systems in *in vitro* cell–cell or *in vivo* systems (54).

Another variant of this technique consists in the use of anti-TCR- (or anti-BCR-) coated latex beads to induce the formation of cell-bead conjugates in suspension (18, 55, 56). These beads, as lipid bilayers, may allow the use of costimulatory agonists (i.e., anti-CD28 for T lymphocytes, anti-CD40 for B lymphocytes) and recombinant proteins such as ICAM-1. Indeed, the use of beads allows lymphocytes to establish a stimulatory contact with a surface more similar to the ellipsoid geometry of an immune cell than the planar lipid bilayer (56).

A more sophisticated variant included in this group of approaches allows to enhance the spatial and temporal control of MTOC repositioning (57) and circumvents the background MTOC polarization issue (see above). This technique consists of the focused activation of a photoactivatable peptide–MHC bound to glass surfaces and has been successfully used to analyze the contribution of the gradient of the second messenger diacylglycerol (DAG) and its negative regulator DAG kinase α to the polarized traffic of lytic granules in CTLs (57, 58). However, this approach suffers, as all the coverslip-based protocols, from the fact that the engaging entity is a lipid laying on a glass-surface and not a “real” cell.

### T Cell-APC Model Systems

#### Conjugates of Jurkat T Cells With Superantigen-Coated Raji B Cells and Human Primary T Lymphoblasts With APCs

For the first approach, Jurkat T cells are challenged with Raji B cells coated with Staphylococcal enterotoxin E (SEE) superantigens (59). This approach can be done in cultured cells in suspension but also using Raji B cells attached to fibronectin-coated coverslips to image the early stages of IS formation [i.e., Supplementary Video 1 in Ref. (36)]. A good number of advantages include: easy handling, well-established human cell lines, feasibility of multiple transfection, availability of Jurkat mutants (60), appropriate kinetics of polarization, valuable model for T cell signaling (60), and the fact that real cell–cell conjugates are imaged. In addition, early signals but also T cell-derived late responses such as IL-2 secretion, and activation-induced apoptosis (19, 36) can be easily measured and/or imaged [Supplementary Material in Ref. (19)] and correlated with trafficking events. Despite of a good number of publications using this model, there are drawbacks related to the use of transformed cells that may lack or overexpress adhesion and accessory molecules typical of primary cells. A certainly more physiological alternative consists in the use of polyclonal, mitogen-activated human T lymphoblasts, or alloreactive human T cell clones of known specificity. Synapses formed by T lymphoblasts with SEE or SEB, Staphylococcal enterotoxin B (SEB) superantigens-coated Raji cells may be used for imaging although a low proportion (20–30%) of human T lymphoblasts harbor the superantigens-interacting Vβ TCR (61). Another option is to polyclonally stimulate T lymphoblasts with anti-TCR bound to lipid bilayers. However, several drawbacks inherent to the use of human-derived samples (such as the presence of potential pathogens), availability of healthy donors, variability in activation responses, requirement for re-stimulation of clones, and low transfection efficiency—among other facts—preclude the extensive use of these models for imaging.

#### Primary (*In Vitro* Stimulated or *In Vivo* Primed) T Cells From TCR-Transgenic Mice

This approach circumvents the problems derived from the use of tumor cells, thus constitutes a fair approach to a more physiological scenario. With the appearance of lentiviral and retroviral gene transduction, the expression of fluorescent chimeras is feasible and these models gained in flexibility (17). However, there is often certain degree of variability (kinetics of activation, expression levels of surface receptors, etc.) that makes this approach somewhat more difficult than the Jurkat–Raji model. Several models can be used with primary T lymphocytes in mice. These models have the additional advantage that the stimulatory peptides or superantigens can be injected *in vivo* to prime immune responses (62). In addition, since all the transgenic lymphocytes express the same TCR with known specificity in quite homogeneous levels, an increase in the efficiency of conjugate formation should be expected when challenged with the specific antigen. A good number of papers on IS imaging have been published by using transgenic murine lymphocytes expressing the ovalbumin (OVA) peptide-specific OT-I TCR (63), which recognizes an OVA peptide. This transgenic TCR is positively selected during thymocyte development in CD8+ CTLs. Thus, OT-I constitutes a good model in the context of CTL polarization and there is a good number of publications arising from this model (17, 64). In addition to OT-I, transgenic murine lymphocytes expressing the influenza NP68 peptide-specific F5 TCR (65), have been successfully used to study traffic in CD8+ CTLs (55). This model also allows for *in vivo* priming of mice with the antigen (62). When the secretory traffic of cytokines in Th synapses needs to be studied, TCR-transgenic lymphocytes positively selected to CD4+ cells (i.e., expressing the transgenic cytochrome C peptide-specific c5.c7 TCR) (2, 13, 31) have been successfully used for imaging. In all these models, potential effects of transgenic expression of the TCR and positive selection during T cell development should be taken into account.

In addition, superantigens models comparable to the described Jurkat–Raji (SEE) human model may also be used in non-TCR-transgenic mice. For instance, SEB superantigens bound to syngeneic APC (i.e., mouse lymphoma EL-4 cells) can be used in mice harboring the appropriate MHC background to challenge a considerable proportion of T lymphoblasts (around 30%, those lymphocytes bearing Vβ3, Vβ7, or Vβ8 TCRs) (36, 66). Caution needs to be taken since synapses made by either CTLs or Th can be simultaneously formed and imaged [Supplementary Video 8 in Ref. (36)] unless a previous separation of CD4+ and CD8+ lymphoblasts is achieved. These superantigens models also allow *in vivo* priming of mice (62). One caveat to consider: although trial-and-error scanning of superantigens-pulsed APCs by T lymphoblasts will indeed be imaged, not all lymphocytes will render mature synapses and this will diminish the frequency of productive, mature synapses.

#### Vertical Cell Pairing (VCP) Device

Observation of lateral cell–cell synaptic conjugates made using the cell models described earlier faces important imaging limitations. Since most conjugates obtained in the classical end-point studies using fixed T lymphocytes and APCs lie parallel to the focus plane, the IS interface lies perpendicular to the plane of focus along the *Z*-axis (Figure 1). Thus, sequential *Z*-stacks need to be imaged to 3D-reconstitute the entire synapse. This is an important caveat for *in vivo* imaging of IS, since conventional confocal microscopes are too slow for 4D imaging (*X, Y, Z, T*) and, in addition have, as already mentioned earlier, a poor resolution in the *Z*-axis. To circumvent all these issues, clever “parking and pairing” devices have been developed by some researchers to study NK synapse (47). The microfluidics contained in this device enhanced the frequency of conjugate formation and induced the positioning of the IS on a horizontal imaging plane, which was the ideal focal plane for IS imaging. Thus, the inherent poor *Z* resolution of microscopes, lack of synchronicity and low frequency of IS interactions can be all together solved by alignment cell devices as VCP. Using these devices, the authors were able to register high-resolution images of lytic granules converging on the IS, simultaneously to actin reorganization (47), in living NK cells forming synapses with target cells. No reports on super-resolution microscopy of T lymphocytes using these devices have been published to date. Drawbacks to consider include that these microfluidic devices are not easy to produce and have not been commercialized to date and therefore are not easily accessible to researchers.

#### Optical Tweezers

For this clever approach, which constitutes an interesting alternative to a physical “parking and pairing device” described earlier, an optical trap was used to place the APC (i.e., Raji cells pulsed with SEE) such that the initial point of contact with the Jurkat cell is opposite from the location of its MTOC, visualized by the expression of GFP-tagged centrin (46). This approach allowed to dynamically image the complete process of MTOC repositioning, avoiding background MTOC polarization. As a drawback, this is a somewhat technically difficult approach and for some unclear reason the speed of MTOC repositioning obtained in Th lymphocytes and Jurkat–Raji conjugates (46) is quite high (less than 1 min to complete) when compared with that obtained using the same cell models without optical trap (several minutes up to few hours) (67, 68). Similarly, by using optical tweezers to manipulate live-cell conjugates and orientate the synapse in the imaging plane of a laser scanning confocal microscope, some authors have imaged protein organization at living NK cell-target cell and T cell-APC immune synapses with high speed and high resolution (69). Optical tweezers have been used in combination with 3D-SIM (see below) to study polarization of lytic granules in NK IS (70).

#### Acoustic Trap

An interesting alternative to physical and optic pairing devices that was used to facilitate, synchronize, and image the NK-target cell synaptic interactions into different wells from multiple well plates, using laser scanning confocal microscopy (LSCM), and also allowed to analyze the heterogeneity in the NK cytolytic response toward tumor target cells (29, 71). The technique allowed en face projections of the intercellular contact (cell interface), but since the formation of conjugates was somewhat forced, this probably may interfere with the establishment of both exploratory/labile and specific/stable contacts that occur between the effector and the target cells. To the best of our knowledge, this application has not yet been used in T lymphocyte synapses, probably because of this caveat.

## Image Capture and Processing

To properly image an IS (as in any fluorescence microscopy technique) a compromise among spatial resolution, temporal resolution, and improved signal-to-noise ratio (SNR) is required (72). Apart from these factors, it is necessary to circumvent the photobleaching and cytotoxicity issues inherent to any living cell imaging (72). Exhaustive reviews have been recently published explaining and comparing the more relevant fluorescence microscopy techniques in cell biology (49, 72), as well as their main features (temporal and spatial resolution) and advantages (72, 73). Please refer to these excellent reviews for additional details, since it is out of the scope of this review to deal with these technical issues. Therefore, we will focus in the most relevant details of these techniques that have been used specifically to image the IS and some representative examples [please note that Table 2.1.2 in Ref. (72) has been modified accordingly in Table 1].

**Table 1:** Comparison of microscopy techniques used for IS imaging.

	Technique	*XY* resolution^a^	*Z* resolution^a^	Temporal resolution	Imaging depth	Usability	Cost^b^	SNR^c^	Photobleaching/phototoxicity^e^	Reference
	Wide-field (WFM)	Diffraction limited (≈200 nm)	Poor (usually <1 µm)	Best (ms/frame, signal limited)Good for 3D imaging	Typically <30 μm	Simple versatile	1	High	Best (usually μW distributed over large imaging field)	(19)(36)

	+Deconvolution	(≈100 nm)^d^	(≈500 nm)^d^					Better (deconvolution)		

	Total internal reflection microscopy (TIRFM) TIRFM–structured illumination microscopy (SIM)	Diffraction limited but low background	Best but only first 200–300 nm	Good (ms/frame, signal limited)	Worst <300 nm	Good	2	High	Better	(74)(75, 50, 52)

	Laser scanning confocal (LSCM)	Diffraction limited to nearly 2× diffraction limit (airy scan)	Good (<700 nm)	Varies with scanner type (typically 1–30 fps) medium	Better (<100 μm)	Complex but most versatile	2–7	Moderate	Can be bad (μW of power focused to spot)	(67)(47)

	Multi-point/slit confocal spinning disk	Diffraction limited	Good (slightly worse than LSCM)	Good (ms/frame, signal limited)	Typically <50 µm	Better	2–4	Moderate	Better (usually lower excitation flux density than LSCM)	(68)(17)(46)

	Two-photon fluorescence microscopy (TPFM)	Diffraction limited	Good (slightly less than LSCM)	Varies with scanner type (typically 1–30 fps) medium-slow	Best (hundreds of μm)	Complex	3–7 with 1 pulsed laser	Moderate	Can be bad (μW power focused to spot but only eliciting fluorescence from the focal plane)	(76)(27)

	(SIM)	Diffraction limited	Good—usually worse than LSCM	Typically 1–10 fps slow	Typically <30 μm	Complex	1.5	Moderate	Good (varies with number of images needed)	(70)(77)

SR	Super-resolution-SIM (SR-SIM) (3D-SIM)	Super-resolution to at least 2× diffraction limit with deconvolution	To 2× diffraction limit with deconvolution	Good (can be ms/frame with iSIM, signal limited)	Typically <10 µm, iSIM < 50 µm	Better (if deconvolved)	4–9	Moderate	Typically good	(52)

	Stimulated emission depletion (STED) microscopy	Super-resolution (<70 nm)	Same as LSCM or <100 nm with axial phase plate	Varies with scanner type (typically 1–30 fps) medium	Typically <50 μm	Complex	6–10	Low	Worst (second beam with many μW of power)	(78)(79)

	Single molecule localization microscopy (SMLM)Photoactivated localization microscopy (PALM)Stochastic optical reconstruction microscopy (STORM) SMLM-3D	Best super-resolution (<30 nm)	Can be ~100 nm or less	Worst requires thousands of imagesVery slow	Typically <a few μm or <200 nm	Complex and requires pos acquisition processing	3–4	Low (noisy if marker density too low)	Varies with technique, can be harsh, typically requires thousands of images	(74)(80)(81)(82)(83)

	Light sheet fluorescence microscopy (LSFM)	Diffraction limited but typically low-mid level numeric aperture (NA) lenses are used	Good depends on light sheet thickness and objective NA	Best for 3D imaging	Best (hundreds of μm)	Better but requires calibration	2–6	High	Best for 3D (*Z*-stack) or 4D (*Z*-stack over time) imaging	(84)

SR	Lattice light sheet fluorescence microscopy (LLSFM) with SIM	Super-resolution to 2× diffraction limit with deconvolution	Super-resolution to 2× diffraction limit with deconvolution	Best for 3D imaging	Typically <20 μm	Complex	2–6	Moderate	Best for 3D (*Z*-stack) or 4D (*Z*-stack over time) imaging	(85)(17)(78)

*Green boxes are best in category, red are worse, modified from reference (72). SR indicates the Super-resolution techniques, based in the axial, *Z* resolution as the main criteria*.

*^a^For a superb diagram of 3D resolution data obtained from cell interiors for all the microscopy techniques please refer to in Ref. (49)*.

*^b^Relative cost is shown, 1 equals to 100,000$ approximately*.

*^c^Signal-to-noise ratio (SNR) is the intensity of the signal of interest divided by the variance in the signal due to noise*.

*^d^Deconvolution can be applied to the different techniques. Data for WFM was obtained from SVI web: https://svi.nl/ResolutionImprovement*.

*^e^This is a general assumption; the photobleaching/phototoxicity issue has a large dependence on the sample preparation, not only on the technique used. Reproduced with permission from reference (72)*.

### Conventional Wide-Field Fluorescence Microscopy (WFM)

WFM combined with image deconvolution after acquisition (Table 1) is still a powerful approach followed by a considerable number of researchers. Not only economic reasons substantiate this choice; the poor resolution in *Z*-axis (the most important drawback of this technique) can be solved in part by using post-acquisition image deconvolution. Deconvolution is a computational image processing technique that can improve image resolution and contrast (72) up to two times, down to 150–100 nm in *XY* and 500 nm in *Z*-axis (https://svi.nl/Deconvolution) (Table 1). Deconvolution requires the knowledge of the idealized or measured point spread function (PSF) of the microscope and the imaging technique used. Deconvolution requires the acquisition of specific images of the sample, whose number and characteristics are usually determined by the method used for imaging. User has to image the sample following these conditions for a correct deconvolution process and this requires certain expertise. Often the metadata [lens numeric aperture (NA), λ] necessary to generate an idealized PSF are contained in the image file and directly loaded by the deconvolution software. Pros: flexibility in a wide number of fluorochromes that can be simultaneously recorded, rapid and sensitive image capture using last generation, highly sensitive, wide field, and rapid scientific complementary metal-oxide semiconductor cameras, improved SNR after image deconvolution and easy training of users. Cons: blurred raw images (bleed-through of out of focus light) before deconvolution, but significantly improved SNR ratio in de-blurred images after deconvolution, enhancing resolution and maintaining the benefits of WFM. Almost all the imaging software available contain deconvolution algorithms, but the state-of-the art, choice software is provided by SVI (Huygens) that includes several optical options [WFM, LSCM, and stimulated emission depletion (STED) microscopy] specific for the different techniques. Among all these techniques WFM is, by far, the microscopy technique that improves most after deconvolution. One example of the power of deconvolution applied to epifluorescence videos on the polarized traffic of MVBs at the IS (19) is provided:

(video before deconvolution) https://www.youtube.com/watch?v=mID0m3usQOQ
(video after deconvolution) https://www.youtube.com/watch?v=Aj0vPj6WAII

### Total Internal Reflection Microscopy (TIRFM)

This technique is particularly useful to study the degranulation processes occurring on a coverslip or a functionalized surface/lipid bilayer (50, 52) due to its intrinsic low background and high SNR (Table 1). However, the technique is somewhat limited since only an illuminated homogeneous surface can be used to stimulate the cells. Another major disadvantage is that TIRFM excludes intracellular structures (i.e., secretory vesicles) or molecules located beyond a small *Z* distance from the coverslip (>200–300 nm). Thus, their initial movement from distal subcellular locations cannot be imaged, although their final movement approaching the IS membrane can be properly imaged. One possibility to avoid these last limitations is to simultaneously combine TIRFM and WFM (57), although “real” cell–cell synapses cannot be imaged. Another recent and interesting option has been developed by using TIRFM-SIM in combination with STED (see below), to show that local dynamism of filamentous actin rearrangements at the nanometer scale is required for cytolytic function of CTLs and NK cells through facilitating degranulation (75). The quality of the imaging obtained in this context is remarkable.

### Laser Scanning Confocal Microscopy (LSCM)

Still one the techniques of choice due to its versatility (availability of flurochromes, etc.), although its low temporal resolution can be a problem when ongoing synapses and derived early signaling need to be imaged, in particular with CTL synapses. However, a considerable number of publications use this particular technique since confocal microscopes are standard core equipment widely used in biology institutes. In addition the quality of certain synapse imaging, both for end-point and time-lapse analyzes is unbeatable (67).

### Spinning-Disk Confocal Microscopy

Conventional LSCM uses a laser to illuminate a single point on the sample and scans across to generate the image. Because it takes several seconds to generate each optical section, events that occur in milliseconds or seconds/minutes range (i.e., intracellular calcium rises, MTOC translocation to the IS) cannot be visualized *via* LSCM. In addition, while the image is being assembled point by point, the laser hits the complete thickness of the specimen frequently but only detects a single focal plane, leading to cytotoxicity and bleaching of the fluorophore (Table 1). Thus, the “efficiency” of excitation and the emission detection is quite low. However, in spinning-disk confocal microscopy, the excitation light is split through a disk containing multiple micropinholes, allowing for several points on the sample to be imaged at the same time. This allows for high-speed confocal imaging and reduced toxicity because of decreased repeated illumination, but also increases the temporal resolution by a factor of 10- to 100-fold when compared with LSCM. This technique has been successfully used for rapid 4D imaging of MTOC translocation to Jurkat–Raji synapses (46, 68). For rapid CTL synapses, the even faster lattice light sheet fluorescence microscopy (LLSFM) (see below) appears to be even better. Pros: rapid acquisition, good image quality, and reduced photobleaching.

### Two-Photon Fluorescence Microscopy (TPFM)

By far, this is the most appropriate technique for *in vivo* imaging since the high penetration of the excitation light allows to deeply penetrate into specimens such as whole lymphoid organs and organoids (76). However, some other features are not so appealing when compared with other techniques (Table 1), thus few publications analyzing intracellular traffic use this technique. However, it has allowed to track migrating T cells, to image synapse formation by CD4+ lymphocytes and to study subcellular dynamics of T cell immune synapses in lymph nodes (27, 76).

### Light Sheet Fluorescence Microscopy (LSFM)

A faster method to generate optically sectioned images when compared with SIM and LSCM, while cytotoxicity and bleaching are reduced. A thin sheet of light produced by a cylindrical lens excites a single plane of the sample, and the emission from the whole plane is collected with a CCD camera that is orthogonal to the excitation light. Because the image is not assembled as points (as in LSCM), imaging is fast and there is a good correlation between illumination and detection of fluorescence, thus decreasing phototoxicity and bleaching (72). Strictly, in its original basic version LSFM is not able to achieve super-resolution images, but depending of light sheet thickness and NA of the lenses, allows better *XYZ* resolution than LSCM and spinning-disk microscopes. LSFM has been used to image immunologically relevant tissues such as lymph nodes (84), but the implementation of LLSFM (see below), which provides further improvement, has overtaken LSFM as a choice technique. LSFM allows rapid 3D time-lapse with reduced photobleaching.

### Super-Resolution (SR) Techniques

Images from the microscopes described earlier are limited by the diffraction of light to a maximal *XY* resolution of about 200 nm (blue light). Although indeed coarse and valuable information can be extracted from these images, they are unsuitable for the study, when required, of nanoscale molecular changes and submicron organization (i.e., detailed interactions of myosin with actin and TCR microclusters, etc.). To circumvent this, a family of super-resolution fluorescence microscopy techniques that achieve resolution beyond this diffraction limit (labeled with a vertical line as “SR” in Table 1) has been developed during the last years. It is important to remark that super-resolution techniques have an important cost: acquisition times are often too long for fast biological processes such as those occurring at the IS, fluorescence probes are limited and 3D imaging is still challenging for single-molecule localization microscopy (SMLM) techniques. This fact, together with the possibility of artifacts (see below), causes that SR techniques should be preferred only when diffraction-limited methods cannot provide the information required (73).

#### SIM and Super-Resolution SIM (SR/3D-SIM)

This technique encompasses a range of techniques based on sample being illuminated with spatially structured light, generating images with high spatial frequency information, which are mathematically reconstructed into a super-resolution image that has twofold better resolution than a conventional WFM image. This technique uses potentially less light, achieves higher temporal resolution, costs less and works well with many more fluorophores than, for instance, STED and SMLM techniques (see Table 1). Part of the improvements of this technique involves also super-resolution (SR-SIM), also called 3D-SIM. This enhances 2-fold the SIM spatial resolution and improves also the temporal resolution up to 100-fold. Superb images of cortical actin reorganization and lytic granules at the IS in NK cells have been published (70). Cons of conventional SIM include low speed and the computational time necessary to obtain the final image as well as the susceptibility to artifact generation during the image reconstitution processes (73).

#### Stimulated Emission Depletion (STED) Microscopy

This super-resolution technique enhances resolution by approximately one order of magnitude when referred to diffraction-limited approaches such as conventional LSCM (Table 1). Sample is scanned with two overlapping lasers: the first one excites and the second depletes the fluorophores, minimizing the volume of detection. Thus, PSF undergoes shrinking by STED leading to an enhancement in *XYZ* resolution. The level of resolution is only surpassed by SMLM (Table 1), but since STED is based on optical design it does not need complex post-acquisition processing as compared with SMLM. STED does not obligatorily entail image processing so it is praised as less susceptible to artifacts than other SR techniques (SR-SIM and SMLM) (73). Since STED acquisition is faster than SMLM, it can be more versatile for live-cell and 3D imaging, although it is significantly slower than spinning-disk microscopy or LLSFM. However, there is a limitation in the number and various fluorochromes that can be used, the power of the lasers induces photobleaching and toxicity and the equipment has a high cost. This technique has been used to image actin cytoskeleton rearrangements and lytic granules in NK synapses (79) and T lymphocytes stimulated on modified glass surfaces (78).

#### SMLM, Photoactivated Localization Microscopy (PALM), and Stochastic Optical Reconstitution Microscopy (STORM)

In practice, SMLM techniques provide the highest spatial resolution available (less than 20 nm in *X*/*Y* and less than 100 nm in *Z*) (Table 1). PALM and STORM use confined (to a higher precision than the diffraction limit), single fluorophore molecule imaging of many cycles of photoactivation and photoconversion to reconstruct a pattern of fluorophores too crowded for single fluorophore imaging. This is achieved by using a limited number of fluorescent probes (photo-switchable fluorescent protein for PALM and conventional synthetic dyes for STORM), which constitutes a caveat. Another important drawback is the large number of images that are required to capture to fully define a single super-resolution image. In addition, complex reconstitution post-acquisition algorithms are necessary to create the final image. Although there are emerging, fast algorithms, processing time and big data storage remain as important concerns, limiting the application of these techniques to fixed samples. Thus acquisition time is a limiting factor and therefore living image applications and 3D/4D experiments are still technically challenging. In addition, because of topological constraints and the difficulty of imaging the irregular surface between two cells, SMLM imaging has been performed using various model systems in which the activating surface can be visualized in a single plane. For this reason these studies have been performed in immobilized surfaces and glass-supported lipid bilayers, and have not been extended to cell–cell conjugates yet. This limitation has motivated the development of a number of three-dimensional (3D), single-molecule imaging techniques, which allow for complete sampling of the protein distribution with high precision in all dimensions. In this context, the development of the double-helix point spread function is an example of a rotating PSF whose intensity distributions rotate as they propagate along the optical axis, allowing access to a significantly larger depth of field (~4 μm). This technique allowed to accurately visualize large-scale receptor reorganization of the membrane of whole T cells and single-particle tracking analyzes in living cells (83).

Using STORM/PALM, it has been shown that early TCR stimulation on glass coverslips recruits lck into clusters (80); using PALM it has been shown that TCR and linker activation T cells (LAT) exist in separate membrane domains (protein islands) in resting T lymphocytes and that these domains concatenated after T cell activation (81). Recently, SMLM has been extended to 3D (82) but is still far from being implemented as a standard technique. In this 3D-SMLM example, the authors explore LAT clustering at the T cell IS in 3D using iPALM and quantitative Bayesian cluster analysis. They show that the increase in LAT clustering observed in 2D results, at least in part, from the recruitment of LAT vesicles to the IS from a deep intracellular pool, distinct from the membrane population (82). More generally, with 3D-SMLM becoming a regularly used tool to address biological questions, the development of an accurate and robust 3D cluster analysis method, as presented by these authors, is an important and necessary advance.

#### Lattice Light Sheet Fluorescence Microscopy (LLSFM)

This state-of-the-art technique is an improvement of rapid LSFM and allows for very rapid 4D imaging of IS (85), in particular rapid signaling and trafficking events in CTL and NK synapses (17, 64). Since the capture in *Z* dimension is extremely fast (10- to 15-fold improvement in temporal resolution when compared with rapid spinning-disk confocal microscopy) and also achieves super-resolution, a 3D full volume information can be imaged each second. In addition, the resolution achieved (threefold finer optical sectioning) allowed to gain a deep insight into actin cytoskeleton reorganization and to establish spatiotemporal relationships between actin reorganization and secretory traffic in CTLs synapse (17). Pros: very rapid acquisition, good image quality, very good 3D sectioning-reconstitution, optimized for live-cell imaging. Cons: complex and expensive equipment.

## Potential Future Developments in the Field

The particular limitations of some of the described techniques can be avoided by applying several of the techniques in the same synapse model [i.e., WFM and LSCM (19), 3D-SIM and LSCM (70), STED and LSCM (79), STED and LLSFM (78), TIRFM-SIM and SMLM-STORM (74), STED, SIM, and TIRF-SIM (75)]. This is an immediate possibility that indeed allows to circumvent the limitations of the current techniques and to synergize in solving nascent biological issues. Alternatively, but not exclusively, combining several techniques when possible in the same experimental setup (i.e., photoactivation, TIRFM and WFM) (15, 57), and/or developing new “smart,” user-friendly instrumental designs (86) and new analytical approaches [i.e., super-resolution radial fluctuations (87)] will allow to tackle the present and future biological problems related to IS imaging. One important challenge occurring mainly in SR techniques is the huge amount of data generated; storing and analyzing the data can be a challenge in itself, since some of these microscopes (SMLM, SIM) do not allow visualizing the images during the acquisition and require post-acquisition complex data processing. Thus, the possibility of secondary artifacts at this stage is a reality (73). One possibility to detect artifacts before or after image processing and calibrate the SR instruments consists of the use of appropriate external image standards (73). Unfortunately, they are not available yet and will not likely be commercially available in the near future. Another interesting option to avoid potential SMLM-derived artifacts is to contrast SMLM results with those obtaining by imaging in parallel the same biological sample with a different technique such as SIM (77). In addition, and during the pre-acquisition stage, the generation of new fluorochromes with more photostability, emission colors, and brighter fluorochromes such as silicon-rhodamine SiR dyes (88) will indeed help in the development of super-resolution techniques. Another biologically relevant and immediate area of application of SR techniques includes the study of maturation (formation of ILVs inside MVBs) and secretory traffic of MVBs. Currently, the nano size of MVBs (up to 500 nm) and ILVs (50–100 nm) requires the use of electron microscopy techniques (32) or correlative microscopy techniques (89, 90), which do not allow studies of living cells. MVBs and ILVs thus are ideal candidates to be imaged by SR techniques, particularly 3D-SMLM, in living cells. The nanoimmunology field will indeed benefit in the immediate future of the application of these techniques.

## Conclusion

Some of the strategies we describe here have been shown to be very useful to obtain high-quality, high-resolution and super-resolution imaging of the IS in living cells, including simultaneous imaging of cytoskeleton changes and trafficking events occurring in living cells forming IS. This has allowed establishing correlations among certain early signaling events, cytoskeleton changes and secretory vesicle trafficking. However, some of these tactics require the use of reductionist approaches (at least from the biological point of view) that need to be considered in advance. Other experimental approaches, by avoiding these caveats, have proved to be very useful since they allow 4D, improved SNR imaging of “real” cell–cell synapses (17, 64). However, in some of these approaches the low frequency of conjugate formation and the low probability to image synapses in formation from the beginning (in particular in the rapid synapses formed by CTL) precludes obtaining valuable data without a previously designed, high-throughput cell “parking and pairing” strategy. The drawbacks inherent to certain SR techniques cause that, in general, these techniques should be preferred only when diffraction-limited microscopy methods cannot provide the information required.

## Author Contributions

VC and MI conceived the manuscript and the writing of the manuscript and approved its final content.

## Conflict of Interest Statement

The authors declare that the research was conducted in the absence of any commercial or financial relationships that could be construed as a potential conflict of interest.

## Abbreviations

AICD, activation-induced cell death; ADAP, adhesion and degranulation promoting adapter protein; APC, antigen-presenting cell; CCD, charged coupled device; cSMAC, central supramolecular activation cluster; CTL, cytotoxic T lymphocyte; DHPSF, double-helix point spread function; F-actin, filamentous actin; ILV, intraluminal vesicles; IS, immune synapse; MHC, major histocompatibility complex; LAT, linker activation T cells; LLSFM, lattice light sheet fluorescence microscopy; LSFM, light sheet fluorescence microscopy; LSCM, laser scanning confocal microscopy; NA, numeric aperture; MVB, multivesicular body; MTOC, microtubule-organizing center; NK, natural killer; OVA, ovalbumin; PSF, point spread function; pSMAC, peripheral supramolecular activation cluster; PALM, photoactivated localization microscopy; sCMOS, scientific complementary metal-oxide semiconductor; SEE, Staphylococcal enterotoxin E; SEB, Staphylococcal enterotoxin B; SIM, structured illumination microscopy; SMLM, single-molecule localization microscopy; SNR, signal-to-noise ratio; SR, super resolution; STED, stimulated emission depletion microscopy; STORM, stochastic optical reconstruction microscopy; TCR, T-cell receptor for antigen; Th, helper T; TIRFM, total internal reflection microscopy; TPFM, two-photon fluorescence microscopy; VCP, vertical cell pairing; WFM, wide-field fluorescence microscopy.
